# Biofabrication of Functional Pullulan by *Aureobasidium pullulans* under the Effect of Varying Mineral Salts and Sugar Stress Conditions

**DOI:** 10.3390/molecules28062478

**Published:** 2023-03-08

**Authors:** Katia Van den Eynde, Vik Boon, Rita Caiado Gaspar, Pedro Fardim

**Affiliations:** Chemical and Biochemical Reactor Engineering and Safety, Department of Chemical Engineering, KU Leuven, 3000 Leuven, Belgium

**Keywords:** pullulan, biofabrication, sugar stress, mineral salt stress, FTIR spectroscopy, ^1^H-NMR spectroscopy, molar mass

## Abstract

Pullulan is a linear exopolysaccharide, produced in the fermentation media of *Aureobasidium pullulans*, with a variety of applications in the food and pharmaceutical industries. Pullulan derivatives have growing potential for biomedical applications, but the high cost of pullulan biofabrication currently restricts its commercial use. Better control over pullulan yield, molecular weight and melanin production by altering fermentation conditions could improve the economics. In this study, the effects of sugar and mineral salt stresses on the pullulan production of *A. pullulans* ATCC 42023 were examined in batch processes. The chemical structure of the recovered pullulan was characterized by FTIR and NMR spectroscopy, and the molecular weight distribution was obtained via SEC. Pullulan yield and melanin production varied when the conditions were adjusted, and pullulans with different molar masses were obtained. Higher-yield pullulan production and a lower polydispersity index were observed when CuSO_4_ was added to the fermentation in comparison with the control and with the addition of sugars and other salts. Biofabrication of pullulan under stress conditions is a promising strategy to enhance biopolymer yield and to obtain pullulan with a targeted molecular weight.

## 1. Introduction

Pullulan is a linear water-soluble exopolysaccharide (EPS) produced by the polymorphic fungus *Aureobasidium pullulans*. The linear homopolysaccharide consists of repeating maltotriose units joined together via α(1,6)-glycosidic bonds, and the unique linkage pattern consisting of two α(1,4) bonds and one α(1,6) bond results in some distinctive properties, such as structural flexibility and adhesive ability, as well as the capacity to form fibers and biodegradable films, which are impermeable for oxygen [1,2,3]. The term functional pullulan refers to the wide variety of applications of this polysaccharide in the food and biomedical industry. These applications are growing due to the non-toxic, non-mutagenic, non-immunogenic, biodegradable and edible properties of pullulan. A variety of chemical functionalization strategies can be used to derivatize pullulan, giving it many prospective applications in tissue engineering, controlled drug release and targeted drug and gene delivery [4,5]. However, the current production cost of pullulan is still a limiting factor for large-scale applications, because of its intensive downstream process [6]. 

**Figure 1:**
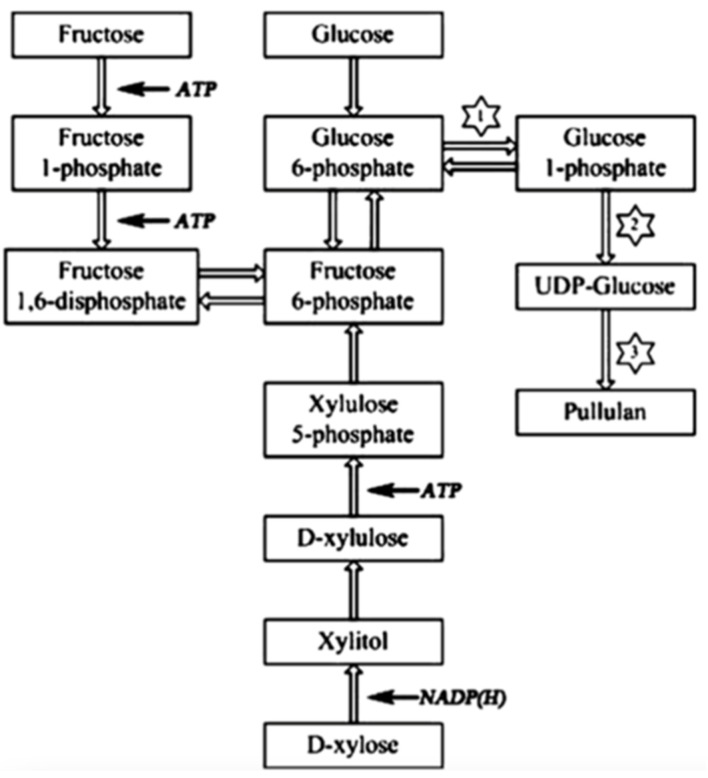
Proposed metabolic pathway of pullulan biosynthesis in *A. pullulans* Y68 by Duan et al. (1 = PGM, 2 = UGP, 3 = FKS). Reprinted with permission from [9] 2023, Elsevier.

This has attracted attention from researchers to optimize the fermentative pullulan production with *A. pullulans* towards higher pullulan yields [1,7,8]. The biofabrication of pullulan is a complex metabolic process governed by environmental conditions [9]. The yield and characteristics of pullulan and, thus, the metabolic pathways of pullulan synthesis are influenced by the composition of the fermentation medium, by the bioreactor design, and by a great variety of environmental conditions, such as pH, temperature and dissolved oxygen. The fermentation medium consists of several nutrients, including carbon sources, nitrogen sources and minerals. Among these cultivation parameters, the nature of the carbon source has proven to have a great influence on pullulan yield and biomass production in batch processes [10,11,12]. Duan et al. [10] proposed a pathway of pullulan biosynthesis, as shown in Figure 1, and reported that more pullulan was produced by the strain *A. pullulans* Y68 in a glucose-containing medium compared to strains cultivated in media containing other sugars due to the higher activity of the three key enzymes, namely α-phosphoglucose mutase, UDPG-pyrophosphorylase and glycosyltransferase. This high enzymatic activity in glucose-containing media results in the continuous production of the pullulan precursor UDPG and, hence, in high pullulan production. Lower pullulan productions were observed in the case of fructose and xylose, probably because of a longer biosynthetic pathway to form UDPG from these sugars. On the other hand, Sheng et al. [11] obtained superior pullulan yields when the strain *A. pullulans* CGMCC1234 was cultivated in a medium containing sucrose as the carbon source because of the formation of kestose by the enzyme β-fructofuranosidase during the early stages of fermentation. The presence of this trisaccharide in the fermentation medium reduces osmotic pressure and enhances cell growth and pullulan production. This means that sucrose is preferred over glucose as the substrate for pullulan production due to the avoidance of hyper-osmotic stress in the medium [11]. The differences in fermentation conditions and in the used strains of *A. pullulans* are a possible explanation for this contradiction.

The focus of most research in this field is the effects of these factors on pullulan yield and molecular weight, but less attention has been paid towards the structure of the EPS produced by the fungus. According to Lee et al. [13], there is however a possibility to modify the composition and structural features of the produced EPS by varying the nature of the carbon source [14]. This study aims to characterize the pullulan obtained from cultures of *A. pullulans* ATCC 42023 grown under mineral salt and sugar stress conditions and attempts to reveal the underlying molecular mechanisms of observed deviations upon sugar and salt stresses. Some metal ions, such as Zn^2+^ and Fe^2+^, play critical roles in biochemical processes and can act as cofactors or inhibitors for certain enzymes, which might be involved in pullulan or melanin synthesis [15,16]. The effects of Cu^2+^ and Fe^2+^ on the synthesis of melanin were evaluated and cultures of *A. pullulans* were grown on different carbon sources to analyze sugar uptake by the microorganism. Certain stress conditions can affect *A. pullulans* towards higher pullulan yields, towards pullulan production of larger molar mass or towards less melanin synthesis, which increase the value of the product and reduce complex steps in downstreaming.

## 2. Results and Discussion

### 2.1. Biofabrication of Pullulan: Effects of Carbon Sources on Pullulan Yield and Biomass Production

**Figure 2:**
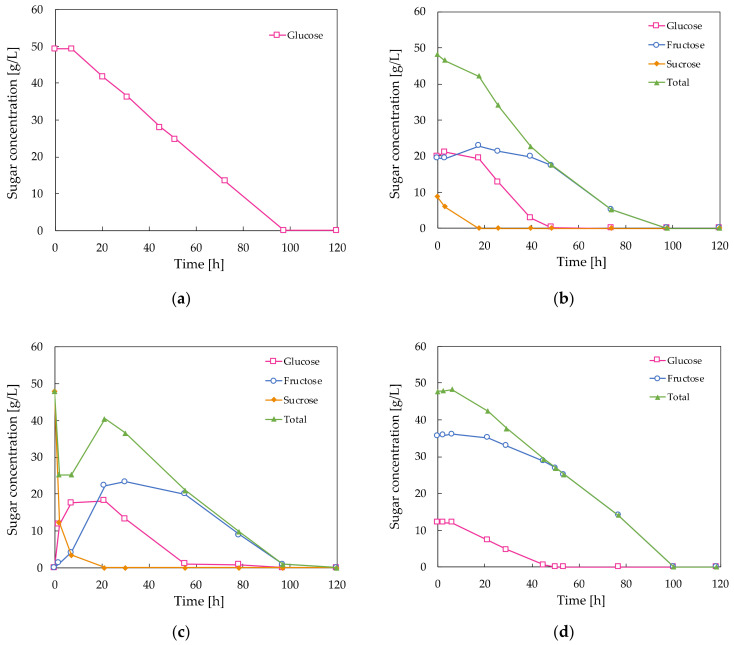
Decaying sugar concentration in the fermentation broths of *A. pullulans* ATCC 42023: (**a**) Medium containing glucose as the carbon source. This is the control medium; (**b**) Medium containing (autoclaved) sucrose as the carbon source. Note that part of the sucrose is already hydrolyzed at time zero due to the heat; (**c**) Medium containing (filtered) sucrose as the carbon source; (**d**) Medium containing a mixture of glucose and fructose in a ratio of 1:4 as the carbon source.

The melanin-producing fungus *Aureobasidium pullulans* was put under sugar stress by alternating the type of carbon source supplemented to the medium, while keeping the total sugar concentration constant at 50 g/L. Glucose was used as the carbon source in the control medium, meaning that the results of other batches were compared to the results of this batch. Three replicates of the control experiments were performed and the coefficient of variation for cell dry weight (CDW) and pullulan dry weight (PDW) were calculated for culture time points of 20, 40, 50, 80, 100 and 120 h (Table S1, Supplementary Materials). The coefficient of variation for CDW and PDW, using glucose as a carbon source and 120 h fermentation time, was 20%. Pullulan production and cell growth are expressed as yields, which are defined as:

(1) $$Y_{\mathrm{JK}}=\frac{\Delta J}{\Delta K^{\prime }}$$

where Y_JK_ is the yield factor, J and K′ are substances involved in the metabolism, ∆J is the mass of J produced and ∆K′ is the mass of K′ consumed. Some frequently used yields are Y_XS_, which is the mass of cells (X) produced per unit mass of substrate (S) consumed; Y_PS_ which represents the mass of product (P) formed per mass of substrate (S); and Y_PX_, which is the mass of product (P) formed per unit mass of cells (X) [17]. The results of the batch fermentations of *A. pullulans* ATCC 42023 with different carbon sources are given in Table 1. After 120 h of fermentation at 30 °C, the highest pullulan concentration was observed when *A. pullulans* was cultivated with a sucrose substrate, while the cell dry weight in these media was lower compared to the glucose-containing control medium, indicating that the carbon flux towards pullulan production is higher when sucrose is used as the substrate as opposed to glucose. The lowest pullulan level was observed when a mixture of glucose and fructose in a 1:4 ratio was supplied as the carbon source, resulting in a lower pullulan yield from substrate Y_PS_ compared to the control. The sucrose carbon source was sterilized once by heat and once by filtration, but this had little effect on the final pullulan and biomass levels in the broths; however, the sugar uptake by the microorganisms was not the same, as displayed in Figure 2. The glucose concentration in the control medium seemed to decay at a constant rate until it was completely consumed after 97 h of fermentation (Figure 2a). The sucrose carbon source was first hydrolyzed into its monosaccharides, glucose and fructose, before it was taken up by the cell. In contrast to the filtered sucrose, part of the sucrose sterilized by heat was already hydrolyzed to glucose and fructose at time zero (Figure 2b). Further hydrolysis takes place in the fermentation medium by means of extracellular invertase enzymes [18]. It is remarkable that the sum of the measured glucose, sucrose and fructose concentrations in Figure 2c did not follow a constantly decaying line, as is the case in the other fermentation broths. This is in accordance with the findings of Seng et al. [11], who concluded that sucrose does not simply hydrolyze to glucose and fructose, but instead kestose is accumulated during the first 24 h of fermentation to reduce osmotic pressure in the extracellular fluid and, consequently, to suppress the inhibitory effects of high sugar concentrations on pullulan production. Hence, this should explain the enhanced pullulan production in sucrose-containing media, but in this study, kestose was not observed in the medium containing autoclaved sucrose, which also resulted in higher pullulan production.

### 2.2. Biofabrication of Pullulan: Effects of Mineral Salts on Pullulan Yield and Biomass Production

Zn^2+^ is an essential metal ion for life because it serves as a cofactor for a large number of proteins. Approximately 9% of the eukaryotic genome encodes proteins that require Zn^2+^ to become functional [19,20]. Therefore, zinc sulphate at a concentration of 0.05 mM was supplied to the control medium. The cell and pullulan dry weight in the Zn^2+^-containing fermentation broth are given in Table 2. The values of both dry weights remained below the final dry weights of the control medium, resulting in an overall pullulan yield from a biomass of Y_PX_ = 1.38 g/g, whereas the final cell and pullulan dry weights were 12 g/L and 16.5 g/L, respectively. This is in agreement with the results of Reeslev and Jensen [21] and Zhang et al. [22], who concluded that ZnSO_4_ increases metabolic flux towards β-glucan production, which is at the expense of pullulan synthesis [22]. 

According to Gadd and Mowll [23], the fungus *A. pullulans* can take up Cu^2+^ intracellularly. Therefore, 0.05 mM of copper sulfate was added to the control medium. Compared to the control medium, the pullulan yield from biomass was almost twice as high because lower cell concentration existed at the expense of higher pullulan synthesis. The pullulan concentration in the fermentation broth amounted to 26 g/L, while the concentration of dry cells only reached a value of approximately 9.8 g/L, as can be seen in Table 2. Pullulan production enhanced by the addition of CuSO_4_ was also observed by Wang et al. [24]. The reasons for enhanced pullulan yield upon CuSO_4_ addition were increased activity of the three key enzymes involved in pullulan synthesis (PGM, UGP and FKS), favored regeneration of energy (high intracellular ATP levels) and an accelerated rate of pullulan secretion from the cell membrane [24]. 

Iron sulphate was supplied to the fermentation medium for the same reasons as Cu^2+^. The cell concentration and pullulan concentration in the fermentation medium are given in Table 2. Similar to the control medium, the cell dry weight reached a value of approximately 14 g/L in this reactor. On the other hand, the pullulan concentration increased to 27 g/L, but did not seem to be stagnant after 120 h. 

Table 2 compares the effect of the different metal ions on the observed yields. It can be seen that the pullulan yield from substrate Y_PS_ of the Fe^2+^-containing medium did not differ much from that of the medium, including Cu^2+^. However, the pullulan yield from biomass Y_PX_ for Fe^2+^ was considerably lower than that of Cu^2+^ because there was a coupling between biomass formation and product formation in the case of Fe^2+^ addition. This indicates that Fe^2+^ could be a cofactor for enzymes that are related to the production of both biomass and pullulan, while Cu^2+^ only increases the activity of pullulan-producing enzymes. 

**Figure 3:**
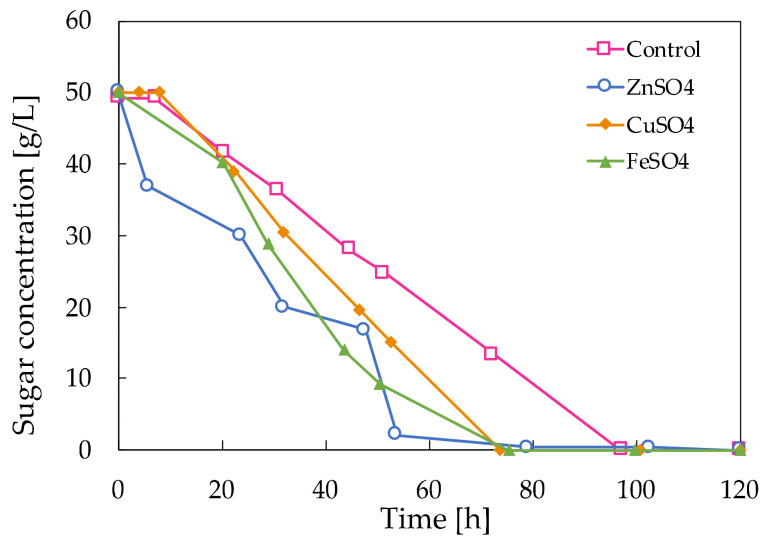
Decaying sugar concentration in the fermentation broths of *A. pullulans* ATCC 42023 with mineral salt stress compared to the control.

In Figure 3, the glucose consumption rate of the control medium is compared to the rate of glucose uptake when Zn^2+^, Cu^2+^ and Fe^2+^ are added to the control medium. The substrate depleted much faster when mineral salts were added compared to the control medium. Hence, as the microorganism is stressed by the presence of the Zn^2+^, Cu^2+^ or Fe^2+^ ions, it consumes the substrate faster compared to the control without the addition of mineral salts.

### 2.3. Biofabrication of Pullulan with Different Molar Masses

**Figure 4:**
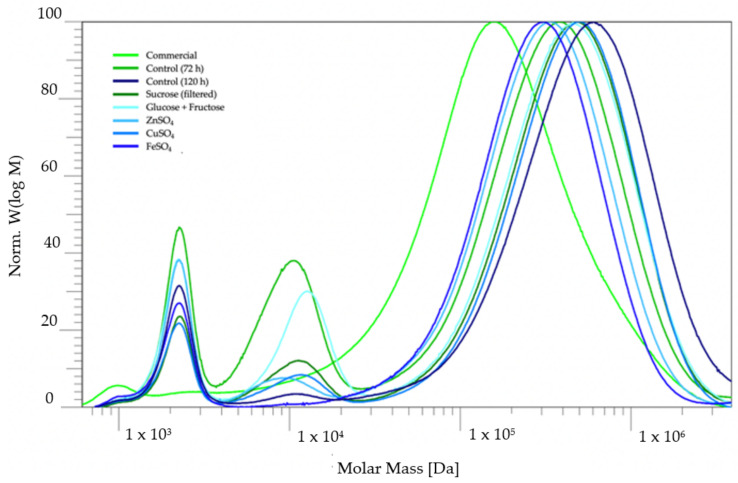
Molar mass distributions of the commercial and experimental pullulan.

The molar mass distributions of the pullulan recovered from the different batches were determined via size-exclusion chromatography (SEC) and compared to the commercially obtained pullulan (Figure 4). The results of the SEC measurements indicate that the molar mass of the experimental pullulan was higher than the molar mass of the commercial pullulan, but it was also observed that the samples of the experimental pullulan possessed a material of low, medium and high molecular weight (M). It is remarkable that the high M part of the distribution shifted horizontally depending on the origin of the pullulan, but the low M fractions were always centered around approximately 2200 Da. The weight-average (M_w_) and number-average (M_n_) molecular weights of the high M fraction are given in Table 3, along with the polydispersity index (PDI) of the high M distribution. 

It is interesting to compare the distributions of the pullulan recovered from the control medium after 72 h and 120 h. The molecular weight of produced pullulan tends to decrease upon prolonged fermentation due to the action of pullulanase enzymes [2]. Looking at the distributions in Figure 4 or at the values in Table 3, the pullulan recovered after 72 h indeed had larger molar masses compared to the pullulan recovered at 120 h of fermentation. The medium M fraction in the distribution of the pullulan recovered at 72 h was also not so pronounced in contrast to the pullulan recovered after 120 h, which is more likely to be hydrolyzed by pullulanases. This could suggest that the medium M fraction originates from the pullulan chains which were cut by pullulanases. 

Looking at the average values of the high M fraction in Table 3, it is noticeable that changing the carbon source from glucose to both sucrose or the mixture of glucose and fructose yielded pullulan of higher molar mass. Additionally, the addition of CuSO_4_ to the control medium resulted in a higher molar mass and a narrower distribution. Zinc sulphate and iron sulphate, on the other hand, seemed to lower molar mass compared to the pullulan from the control medium. However, there was no medium M fraction present in the molecular weight distribution of iron, indicating that Fe^2+^ inhibits the action of pullulanases or the formation of pullulanases, but also affects the pullulan biosynthesis towards shorter chain lengths. On the other hand, the lower molar mass of the pullulan resulting from the zinc-sulphate-containing media could indicate that Zn^2+^ acts as a cofactor for pullulanases.

### 2.4. FT-IR and ^1^H-NMR of Pullulan

**Figure 5:**
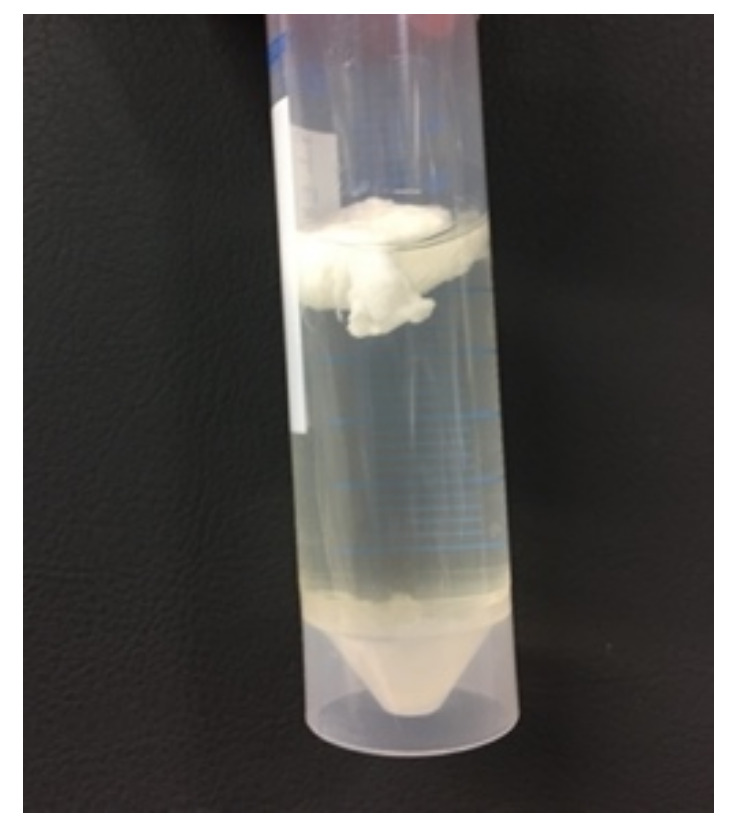
Two types of precipitates were obtained after precipitation in ethanol.

The harvested pullulan was characterized with FTIR spectroscopy. Two different types of precipitates were observed after the precipitation of one volume supernatant into two volumes of ethanol. As seen in Figure 5, one precipitate floated, while the other settled at the bottom. Both precipitates were filtered separately in order to investigate their composition.

**Figure 6:**
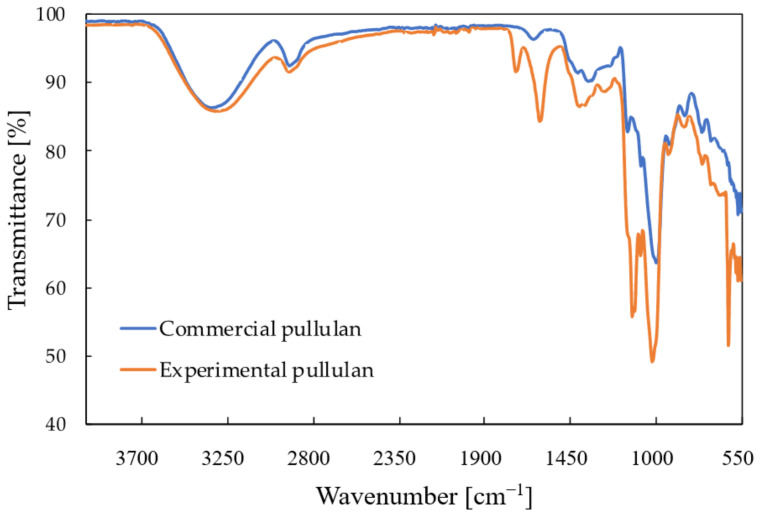
FTIR spectrum of pullulan recovered from the control medium after 120 h (orange) compared to the FTIR spectrum of commercial pullulan (blue).

The FTIR spectra of the top precipitate recovered from the control medium after 120 h is shown in Figure 6, together with the spectrum of commercial pullulan. Table 4 compares the spectral data of the top precipitate to that of the commercial pullulan. It can be concluded that this part of the precipitate indeed consists of pullulan. The spectrum of the control resembles that of the commercial pullulan, but some extra peaks were observed at 1730 cm^−1^, 1100 cm^−1^ and 615 cm^−1^. The spectrum of the precipitate at the bottom also contained two peaks, one at 615 cm^−1^ and one at 1100 cm^−1^, which originated from inorganic phosphates that are also insoluble in ethanol [25]. Those were also visible in the spectrum of the top precipitate; therefore, it can be concluded that some phosphate impurities are present in the recovered pullulan. The peak at 1730 cm^−1^ probably corresponds to the C=O stretching of an aldehyde group, but this functional group is normally not part of the pullulan structure. This peak could be attributed to the presence of impurities with an unknown composition. The FTIR spectra of the pullulan recovered from the other media were all similar to that of the control medium.

**Figure 7:**
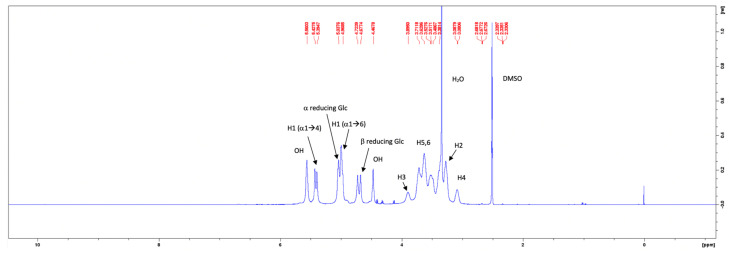
^1^H–NMR spectrum of commercial pullulan.

**Figure 8:**
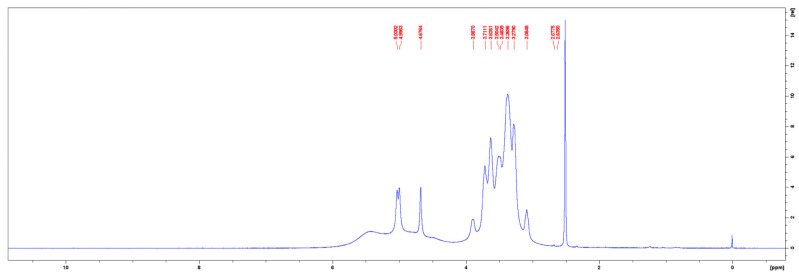
^1^H–NMR spectrum of pullulan from the control medium.

The ^1^H-NMR spectra of the commercial pullulan and the pullulan from the control medium are given in Figure 7 and Figure 8, respectively. The peak at 3.33 ppm in the ^1^H-NMR spectrum of the commercial pullulan originated from the presence of water [26]. The peaks between the chemical shifts of 3.0 and 4.0 ppm correspond to the protons of the anhydroglucose units and the signals of the hydroxyl protons were observed at 4.47 and 5.56 ppm [27,28]. The signals of the anomeric protons appeared at 5.43, 5.39 and 5.04 ppm [29,30]. Note that the peaks at 5.56, 5.43 and 5.39 ppm were contracted to one broad peak at 5.44 ppm in the ^1^H-NMR spectrum of the pullulan obtained from the control medium. Such dissimilarities could be contributed to a much higher molecular weight or a different pH of the solution, caused by impurities such as phosphates. The proton spectrum of the fermentative pullulan between 3.0 to 4.0 ppm is, on the other hand, very similar to the proton spectrum of the commercial pullulan, confirming the purity of the harvested exopolysaccharide. 

## 3. Materials and Methods

### 3.1. Materials

Commercially available pullulan was obtained from TCI chemicals, Tokyo, Japan.

### 3.2. Microorganisms

*A. pullulans* ATCC 42023 was stored at −80 °C in a 20% (*v*/*v*) glycerol solution. Cultures were grown on potato dextrose agar (PDA) Petri dishes at 30 °C for 2 days. The strain on the PDA dishes was maintained at 4 °C and sub-cultured every week. 

### 3.3. Media

The inoculum medium contained 24 g/L potato dextrose broth and was autoclaved for 3 h. Seed cultures were prepared by inoculating a colony grown on a PDA plate into a 250 mL flask that contained 75 mL of the inoculum medium. Subsequently, this was incubated at 30 °C for 20 h with shaking at 200 rpm. After 20 h of incubation, 40 mL of the inoculum medium was centrifuged at 3220 rcf for 15 min at 30 °C and the cells were resuspended in 5 mL of a 0.89% (*w*/*v*) NaCl solution, which was then inoculated in the fermentation medium to start a batch.

The reference medium contained 50 g/L glucose, 2.5 g/L yeast extract, 0.6 g/L (NH_4_)_2_SO_4_, 1.0 g/L NaCl, 5.0 g/L K_2_HPO_4_ and 0.2 g/L MgSO_4_·7H_2_O. The type of sugar and added salts varied according to the experimental scheme. The total initial sugar concentration always remained 50 g/L, and other mineral salts were added at a concentration of 0.05 mM to stress the fungus. Each nutrient was sterilized separately and added together under aseptic conditions. Sterilization by heat was performed in a 160 L autoclave (Laboklav series) and a Nalgene Rapid-Flow filter with a pore size of 0.2 μm was used for sterilization by filtration.

### 3.4. Culture Conditions

Pullulan was produced in a stirred-tank fermenter (Eppendorf Q 869400). The fermenter consisted of a glass vessel with a total volume of 5 L, which contained a working volume of 4 L of the fermentation medium. The pH, temperature, agitation speed and aeration rate in the vessel were controlled automatically by the BioFlo 120 bioprocess controller from Eppendorf. Precise temperature control was provided through a heat blanket and a water-recirculation module, which kept the temperature at a constant value of 30 °C. The pH was measured by a pH sensor (EasyFerm Bio K8 325) and maintained at 5.0 by adding either 4 M KOH or 2 M H_2_SO_4_. Baffles were absent and agitation was provided by two six-flat-blade impellers (diameter = 6 cm), located 4 cm and 12 cm above the bottom of the vessel, respectively. Pullulan production was carried out at an aeration rate of 1 vvm (volume of air per volume of liquid per minute) and a constant agitation speed of 500 rpm. The reference medium batch of glucose was performed in three repetitions to assess variation in CDW and PDW over time (Table S1 and Figure S1, Supplementary Materials). 

### 3.5. Analytical Methods

Residual sugar content in the culture broth was determined by high-performance liquid chromatography (HPLC). Approximately 1.5 mL of supernatant was filtered through a 0.20 μm syringe filter (Thermo Fisher), which was stored at 4 °C. The HPLC analyses were performed under the following conditions: instrument model, Agilent 1200 series; column, BIO-RAD Aminex HPX-87H (7.8 mm × 300 mm); mobile phase, 5 mM sulfuric acid in water; flow rate, 0.6 mL/min; column temperature, 40 °C; detectors, Diode Array Detector (UV/VIS) set at 210 nm and RI detector set to 40 °C; and injection volume, 20 μL.

After 120 h of fermentation, the medium was collected and centrifuged at 3220 rcf and 30 °C for 15 min to remove the majority of cells. The supernatant was decanted from the cells and poured into 50 mL falcon tubes, which were then placed in a water bath for 1 h at 80 °C in order to deactivate extracellular pullulanases [31]. After the supernatant was cooled to room temperature, it was centrifuged again at 10,000 rcf for 15 min at 20 °C to further remove smaller cells and spores. Then, the pullulan was precipitated using two volumes of 99% ethanol per volume of supernatant, and this was left in the fridge at 4 °C for one day. The floating EPS precipitate was torn apart to wash it three times with 99% ethanol, after which it was dried until a constant weight was reached.

The characterization of the purified EPS was carried out using a Perkin Elmer Spectrum 100 FTIR spectrometer system over a range of 4000–550 cm^−1^. A total of 16 scans with a resolution of 4 cm^−1^ were acquired and averaged. NMR spectra were acquired on commercial instruments Bruker Avance 300 MHz and Bruker AMX 400 MHz. All the chemical shifts (δ) were reported in parts per million (ppm) relative to trimethylsilane (TMS) as an internal standard. DMSO-d6 was used as a solvent. The chemical shift of DMSO-d6 was at 2.50 ppm.

Size-exclusion chromatography (SEC) was performed on the pullulan samples to determine the molecular weight distribution. Aqueous pullulan samples of 1 g/L were prepared. The SEC analysis happened under the following conditions: data system, PSS WinGPC UniChrom; columns, SUPREMA precolumn, SUPREMA 1000 and SUPREMA 30; detectors, UV-975 and RI-930; flow rate, 1.0 mL/min; buffer solution, aqueous 0.1 M NaNO_3_/NaN_3_; measuring temperature, 30 °C; and injection volume, 100.0 μL.

## 4. Conclusions

The effects of sugar stress and salt stress on the biosynthesis of pullulan by *A. pullulans* were investigated. Among all the tested sugars, sucrose resulted in the highest pullulan yield from the substrate. The enhanced pullulan production due to the sucrose substrate could possibly originate from the synthesis of a trisaccharide, namely kestose, which lowers the fluid’s osmotic pressure and suppresses the inhibitory effect of high sugar concentrations [32]. ZnSO_4_, CuSO_4_ and FeSO_4_ were separately added to the control fermentation medium to examine the influence of these mineral salts on pullulan biosynthesis. The addition of 0.05 mM CuSO_4_ proved to have the most promising effects because it resulted in more pullulan production and a narrower molecular weight distribution with larger Mw compared to the control, which is beneficial for several applications. The addition of ZnSO_4_ to the control medium decreased both stationary cell dry weight and pullulan dry weight, which could be caused by an increased metabolic flux towards β-glucan production. The resulting pullulan was of lower molar mass compared to the control, suggesting that Zn^2+^ could act as a cofactor for pullulan-degrading enzymes, such as pullulanase. The supplementation of FeSO_4_ increased pullulan production compared to the control, but the efficiency of pullulan production by the cells was lower compared to the cells stressed by CuSO_4_. Our strategy of pullulan biosynthesis yields the production of biopolymers with tailored Mw that in the near future will be further studied in enzymatic processing and controlled drug delivery. 

## Figures and Tables

**Table 1:** Effect of carbon sources on pullulan and biomass production by *A. pullulans* ATCC 42023.

Carbon Source	Glucose (Control)	Sucrose (Autoclaved)	Sucrose (Filtered)	Glucose + Fructose
CDW(g/L)	14	9	10	11.5
PDW (g/L)	20	30	28	18
Y_PX_ (g/g)	1.43	3.33	2.8	1.57
Y_XS_ (g/g)	0.28	0.18	0.2	0.23
Y_PS_ (g/g)	0.40	0.60	0.56	0.36

**Table 2:** Effect of mineral salts on pullulan and biomass production by *A. pullulans* ATCC 42023.

Mineral Salts	Control	ZnSO_4_	CuSO_4_	FeSO_4_
CDW(g/L)	14	12	9.8	14
PDW (g/L)	20	16.5	26	27
Y_PX_ (g/g)	1.43	1.38	2.65	1.86
Y_XS_ (g/g)	0.28	0.24	0.20	0.28
Y_PS_ (g/g)	0.40	0.33	0.52	0.54

**Table 3:** Weight-average molecular weight, number-average molecular weight and polydispersity index values of the high molecular weight fraction.

	M_w_ (Da)	M_n_ (Da)	PDI (-)
Commercial	270 120	77 417	3.49
Control (120 h)	516 180	231 070	2.23
Control (72 h)	750 540	316 680	2.37
Sucrose	581 750	287 780	2.02
Glucose + Fructose	573 240	282 480	2.03
ZnSO_4_	439 230	205 450	2.14
CuSO_4_	578 550	301 130	1.92
FeSO_4_	388 280	185 510	2.09

**Table 4:** Comparison between the FTIR spectral data of commercial and fermentative pullulan.

Assignment	Commercial Pullulan Wavenumber (cm^−1^)	Fermentative Pullulan Wavenumber (cm^−1^)
O-H stretching	3320	3336
C-H stretching	2917	2922
O-C-O stretching	1632	1641
C-O-H stretching	1418	1415
C-O-C stretching	1148	1110
C-O stretching	1018	990
α-D-Glucopyranose unit	857	849
α(1,4)-glycosidic linkage	754	755
α(1,6)-glycosidic linkage	1077	1079

## Data Availability

The data presented in this study are available in Supplementary Materials.

## Supplementary Materials

The following supporting information can be downloaded at: https://www.mdpi.com/article/10.3390/molecules28062478/s1. Figure S1: Cell dry weight and pullulan dry weight over time; Table S1: Statistical analysis of the three glucose reference medium batches.

## Sample Availability

Samples of the compounds are not available from the authors.
