# Supplementary material for: Biofabrication of Functional Pullulan by Aureobasidium pullulans under the Effect of Varying Mineral Salts and Sugar Stress Conditions
Source: Molecules. 2023 Mar 8;28(6):2478. doi: 10.3390/molecules28062478 (PMC10056076; doi:10.3390/molecules28062478)
Supplement: Supplementary file 1 [file molecules-28-02478-s001.zip › molecules-1951545-supplementary.pdf]

molecules

Article

Biofabrication of Functional Pullulan by *Aureobasidium pullulans* under the Effect of Varying Mineral Salts and Sugar Stress Conditions

Katia Van den Eynde, Vik Boon, Rita Caiado Gaspar and Pedro Fardim *

[1] Chemical and Biochemical Reactor Engineering and Safety, Department of Chemical Engineering, KU Leuven, 3000 Leuven, Belgium

* Correspondence: pedro.fardim@kuleuven.be; Tel.: +32-16320970

Three reference medium batches using glucose were performed to assess the statistical variation of cell dry weigth (CDW) and pullulan dry weight (PDW). CDW and PDW were monitored over time (Figure 1). The data is summarized and analyzed in Table S1 and the average and coefficient of variation is for each of the parameters of the three reference batches were calculated.

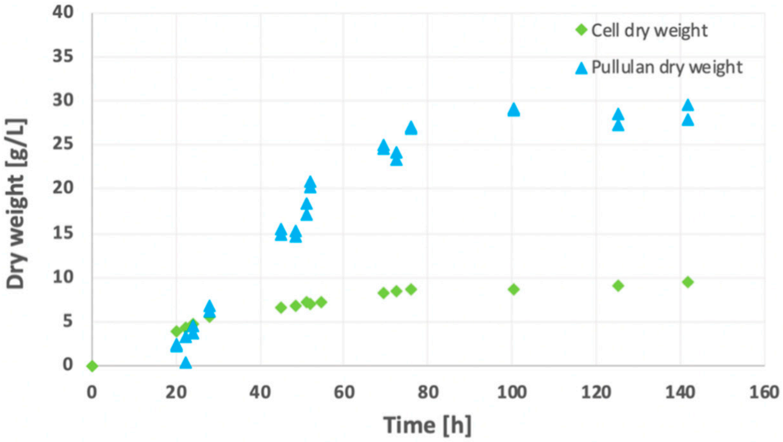

Figure 1: Cell dry weight and pullulan dry weight over time

Table S1. Statistical analysis of the three glucose reference medium batches

Time (h)	Parameter	20	40	60	80	100	120
Batch One	Cell Dry Weight (g/L)	5.3	9.4	13.6	14.2	14.2	14.2
	Pullulan dry weight (g/L)	4.3	9.5	18.2	22	22	22
Batch Two	Cell Dry Weight (g/L)	4.6	6.5	7.5	9.2	9.6	9.8
	Pullulan dry weight (g/L)	3.8	13.5	22.3	27.3	29	28.5
Batch Three	Cell Dry Weight (g/L)	4.4	6.8	9.8	12.3	14.6	14.1
	Pullulan dry weight (g/L)	4.4	6.7	15.1	21.3	22.1	19.3
Average and (coefficient of variation)	Cell Dry Weight (g/L)	4.8 (10)	7.6 (21)	10.3 (30)	11.9 (21)	12.8 (22)	12.7 (20)
	Pullulan dry weight (g/L)	4.2 (7)	9.9 (34)	18.5 (19)	23.5 (14)	24.4 (16)	23.3 (20)
